# Considering Autism in Binge-Type Eating Disorders: Broadening Beyond an Anorexia Nervosa-Focused Model

**DOI:** 10.1007/s11920-026-01719-8

**Published:** 2026-10-09

**Authors:** Lauren Makin, Kate Tchanturia

**Affiliations:** 1 PO59, Department of Psychological Medicine, Institute of Psychiatry, Psychology & Neuroscience, King’s College London, 6 De Crespigny Park, London, SE5 8AF UK; 2 National Institute for Health and Care Research (NIHR) Maudsley Biomedical Research Centre at South London and Maudsley NHS Foundation Trust and King’s College London, London, UK; 3 Department of Eating Disorders, South London and Maudsley NHS foundation Trust, London, UK; 4 Department of Culture and Communication, Aalborg University, Aalborg, Denmark

**Keywords:** Autism spectrum disorder, Eating disorders, Bulimia nervosa, Binge eating disorder, Attention-deficit/hyperactivity disorder, Neurodiversity

## Abstract

**Purpose of Review:**

Research on Autism in eating disorders (EDs) has historically focused on anorexia nervosa (AN), with comparatively limited attention given to binge-type EDs such as bulimia nervosa (BN) and binge eating disorder (BED). This review summarises emerging evidence on Autism in binge-type EDs and highlights key clinical and research implications.

**Recent Findings:**

Emerging evidence suggests that Autism is over-represented in BN and BED, although prevalence appears lower than in AN and research remains methodologically limited. Recent studies suggest that affective and reward-related processes, alongside sensory differences, may contribute to binge-type presentations. Co-occurring ADHD appears particularly relevant and may complicate recognition, clinical presentation, and treatment. Autism also remains under-recognised in binge-type EDs by ED services, where existing adaptations remain largely derived from AN models.

**Summary:**

Current AN-centric models may inadequately capture Neurodivergence in binge-type EDs. More transdiagnostic, mechanism-informed research and treatment approaches are needed.

## Introduction

Autism is over-represented within eating disorder (ED) services and is associated with distinct underlying mechanisms, more complex clinical presentations, poorer treatment experiences, and less favourable outcomes [1–5]. Consequently, there has been increasing interest in adapting ED treatment to better meet the needs of Autistic individuals [6–10]. However, despite increasing recognition of Autism in EDs, the literature remains heavily focused towards anorexia nervosa (AN), with comparatively limited attention given to binge-type EDs, including bulimia nervosa (BN) and binge eating disorder (BED) [1, 11–13]. As a result, current conceptualisations and treatment approaches may be more informed by AN, potentially obscuring distinct mechanisms and clinical needs in binge-type EDs.

**Fig. 1:**
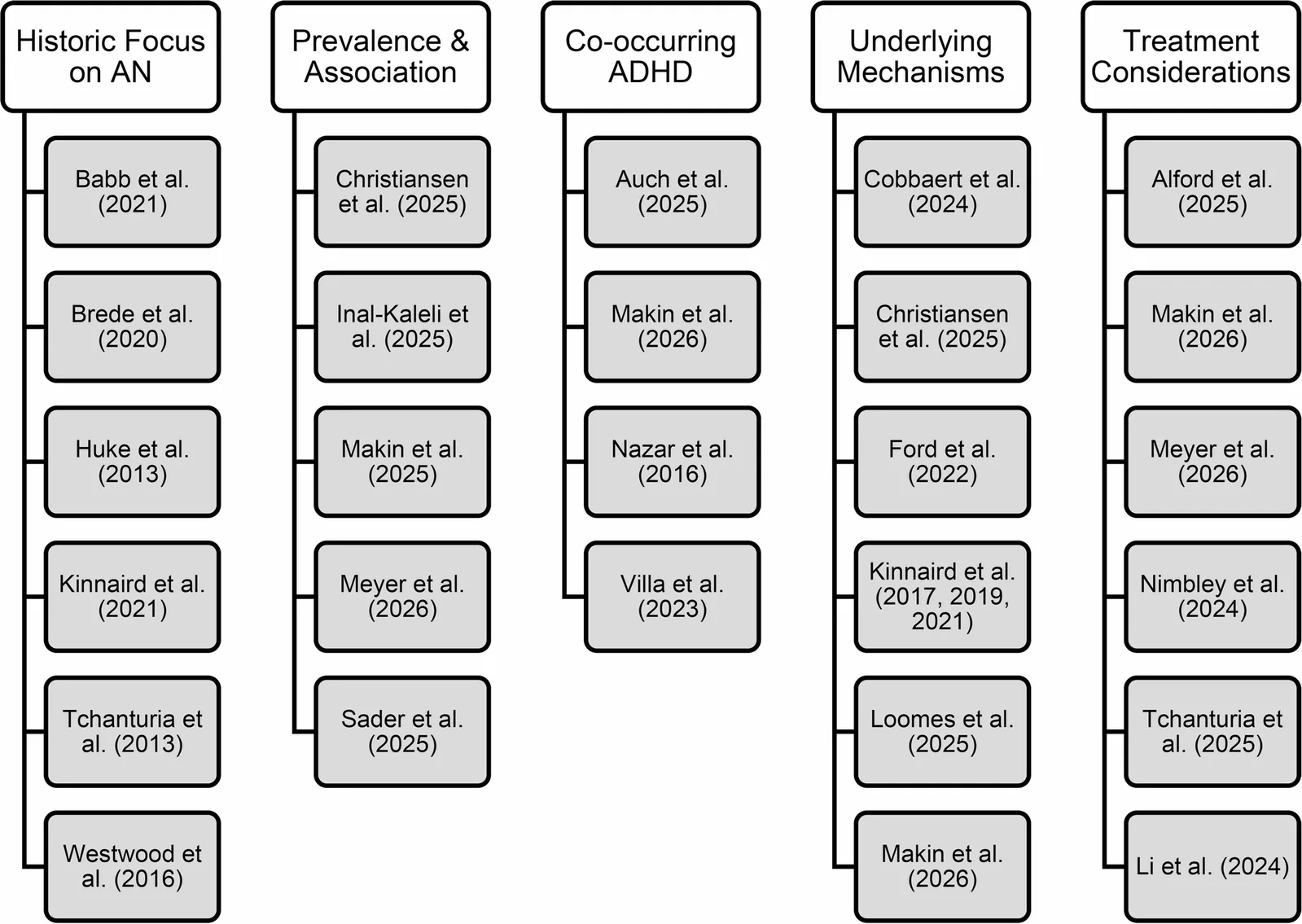
Overview of key thematic areas and representative literature informing this review

Thus, this review summarises emerging research focusing on Autism in binge-type EDs. Given the relative youth and heterogeneity of this literature, this review aims to highlight key studies and emerging clinical issues related to Autism in binge-type EDs rather than providing a systematic review or definitive clinical recommendations. In particular, it examines the prevalence of Autism in binge-type EDs [14–17], proposed mechanisms underlying this relationship [16, 18–20], and the clinical presentation, recognition, and support needs of Autistic individuals with binge-type EDs [17–20] (see Fig. 1). Particular attention is given to qualitative, participatory, and lived-experience research, reflecting growing recognition of the importance of stakeholder perspectives in shaping neuro-affirming care [12, 21, 22]. Language use follows recent neuro-affirming guidelines [23–25]. It is important to note that the existing literature in this area has emerged predominantly from European contexts, particularly the UK, and findings should therefore be interpreted within the context of these healthcare systems, diagnostic practices, and cultural frameworks. 

## Historic Focus on Autism in AN

Until recently, research examining the relationship between Autism and EDs was heavily concentrated on AN [26–28]. Early studies primarily focused on establishing whether Autism was over-represented in individuals with AN, often relying on small clinical samples, self-report trait measures, and debates regarding whether starvation-related effects may artificially inflate Autistic traits [26–29]. Although this work was instrumental in establishing Autism as a relevant consideration within ED populations, it was often methodologically limited and contributed to an AN-centric conceptualisation of Neurodivergence in EDs.

Recent years have seen a notable shift away from prevalence-focused research towards greater exploration of mechanisms and lived experience [30–35]. Studies increasingly examined sensory, cognitive, and social differences as potential contributors to ED development and maintenance in Autistic individuals [30–35]. Qualitative and participatory research also began centring Autistic perspectives, highlighting that eating difficulties may serve functions extending beyond weight and shape concerns, to managing identity and social challenges brought about through social adversity and masking, and challenging assumptions embedded within traditional neurotypical models of ED psychopathology [30–35].

A further major development has been the growing emphasis on treatment adaptation and implementation [6, 7]. The Pathway for Eating disorders and Autism developed from Clinical Experience (PEACE) has been particularly influential in translating research into practice [6, 7, 10]. This introduced Autism screening within UK-based ED services, sensory-informed service adaptations, communication adjustments, alternative meal options, clinician training, and more individualised care planning, and is beginning to be adopted internationally [6, 7]. Importantly, research has increasingly moved beyond describing potential adaptations to evaluating their implementation within real-world clinical settings, with subsequent expansion into child and adolescent services and broader neuro-inclusive care frameworks [36–40].

Most recently, the field has begun to move beyond an exclusive focus on AN. Research is increasingly examining Autism across other ED diagnoses, including avoidant/restrictive food intake disorder (ARFID) and binge-type EDs such as BN and BED, while also considering co-occurring ADHD and broader conceptualisations of Neurodivergence [1, 3, 12, 17–20, 22, 37–39, 41–44]. This expansion has prompted greater attention to binge eating, sensory-seeking, reward sensitivity, emotional overwhelm, impulsivity, executive functioning differences, and diagnostic overshadowing [18–20, 22, 37, 39, 42, 43]. At the same time, researchers have become increasingly critical of methodological limitations within the literature, including over-reliance on trait-based measures, limited use of gold-standard Autism assessments, gender bias, and the under-recognition of Autism in non-AN populations. Together, these developments reflect a broader shift toward more nuanced, transdiagnostic, and clinically applicable models of neurodivergence in EDs.

## Prevalence of Autism in EDs

Autism appears to be substantially over-represented in restrictive EDs. A recent meta-analysis of 22 studies reported that approximately 29% of children and adults with AN scored above the clinical cut-off for Autism on the Autism Diagnostic Observation Schedule (ADOS), widely considered the gold-standard observational assessment [2, 45]. Additionally, in a recent clinical study, 34% of patients with the binge-purge subtype of AN, 32% of patients with the restrictive subtype of AN, and 26% of patients with atypical-AN had elevated autistic traits using the AQ-10 [17, 46]. Similarly, a meta-analysis of 21 studies found that 16% of individuals with ARFID had a diagnosis of Autism [3].

In contrast, the evidence base for binge-type EDs remains more limited and methodologically heterogeneous [1]. Two out of three studies have reported elevated autistic traits in individuals with BN and BED relative to non-ED populations, although these studies relied on self-report measures rather than gold-standard diagnostic assessments [14, 15, 47]. In a recent clinical study, 27% of patients with BN and 17% of patients with BED had elevated autistic traits [17].

Across studies, autistic traits in BN and BED tend to be lower than those observed in AN, although this pattern is not entirely consistent [14, 17, 47, 48]. Large-scale population data provide further support for an association. A recent multi-cohort analysis of over 1.6 million individuals found that those with BN had an increased likelihood of receiving a subsequent Autism diagnosis, although this association was weaker than that observed for AN [16].

Taken together, current evidence suggests that Autism is over-represented in binge-type EDs, albeit to a lesser extent than in restrictive EDs. However, conclusions remain tentative given variability in measurement approaches and the relative scarcity of studies using gold-standard diagnostic tools in BN and BED populations.

## Co-Occurring ADHD

ADHD frequently co-occurs with Autism, which is often known as *AuDHD* [42, 49]. Although ADHD has long been linked to binge eating, higher weight, and addictive behaviours [50–54], it has historically received comparatively less attention than Autism within ED services. However, ADHD is much more prevalent in binge-type EDs than restrictive EDs [1, 55–57], suggesting that co-occurring ADHD may be particularly relevant among Autistic individuals with binge-type EDs [20].

Emerging evidence suggests that ADHD may contribute to binge-type EDs through several overlapping pathways [39]. First, affective pathways appear important, with mood and anxiety symptoms accounting for a substantial proportion of the association between ADHD and EDs [16, 18, 19, 37, 58]. These difficulties may be shaped by chronic experiences of academic, occupational, and interpersonal challenges, as well as pressures to mask ADHD-related differences, which can contribute to long-term feelings of failure or misunderstanding [37, 59–61]. Second, reward-related pathways may also be relevant. ADHD and binge eating appear to share underlying dopaminergic reward mechanisms [58], and ADHD individuals show heightened food responsiveness [62]. Qualitative studies have similarly found that some individuals with binge-type EDs describe binge eating as a form of “dopamine” seeking or stimulation regulation [18, 19]. Finally, sensory and interoceptive pathways may also contribute, as some ADHD individuals report using food to regulate sensory or bodily states, with specific textures, fullness sensations, or eating experiences helping to modulate arousal, attention, or emotional states [18, 19, 37, 63].

ADHD is also associated with increased clinical complexity within ED services. Higher ADHD traits correlate with greater ED psychopathology, particularly binge eating, as well as elevated anxiety and depression [55, 56, 64, 65]. Furthermore, AuDHD individuals may experience particularly high levels of psychological distress and greater work and social functioning difficulties compared to Autistic-only or ADHD-only individuals with EDs [42]. Clinicians have also described ADHD as contributing to impulsivity, emotional overwhelm, elevated self-harm and suicidality risk, executive functioning difficulties, and challenges maintaining daily structure [20, 37]. These differences may also disrupt conventional treatment processes, including attending appointments, completing between-session tasks, or maintaining focus during therapy sessions [37]. Such difficulties may be misinterpreted as disengagement or resistance, rather than manifestations of ADHD-related needs [37, 66]. ADHD adults with EDs also show higher treatment dropout and poorer post-treatment outcomes [67, 68].

This has important implications for understanding Autism in binge-type EDs. ADHD shares several overlapping features with Autism, and in AuDHD individuals it may be difficult to disentangle which traits are contributing to ED onset, maintenance, and treatment challenges [20, 37]. Consequently, some mechanisms currently attributed to Autism in binge-type EDs may also reflect the influence of co-occurring (and often unrecognised) ADHD. This is particularly relevant given that ADHD is not routinely screened for in many UK-based ED services, including some services that do screen for Autism [18–20, 37]. Conversely, more externally visible ADHD traits may also obscure underlying Autism, contributing to delayed recognition [20, 37]. ADHD should therefore be considered alongside Autism when examining Neurodivergence in binge-type EDs.

## Underlying Mechanisms

Several authors have proposed that elevated autistic traits in patients with EDs may partly reflect state-related effects of starvation, prolonged illness, or malnutrition rather than underlying Autism [29, 69, 70]. However, evidence from binge-type EDs, which are generally less likely to involve prolonged starvation or severe underweight than AN, challenges this explanation [14–17, 47]. Supporting this interpretation, a recent scoping review identified longitudinal evidence demonstrating the presence of Autistic traits in childhood, alongside studies reporting little or no association between BMI and autistic traits [71]. Together, these findings suggest that the relationship between Autism and EDs extends beyond acute illness effects. Instead, it has been proposed that shared pathways may contribute to both Autism and EDs, or that Autism itself may directly or indirectly contribute to the development and maintenance of ED psychopathology.

### Affective Mechanisms

Evidence from a large population study suggests that affective processes may play a particularly important role in linking Autism to binge-type EDs; When mood and anxiety disorders are accounted for, the association between Autism and BN is substantially attenuated, suggesting that co-occurring affective difficulties may explain a significant proportion of this relationship [16]. In patients with BN, autistic traits are associated with increased psychological distress [17], as has previously been found in AN samples [72, 73].

This aligns with broader literature indicating that Autistic individuals, particularly in non-affirmative environments, are at increased risk of social alienation, victimisation, masking, and identity-related difficulties, which may contribute to elevated rates of anxiety and depression [4, 18–20, 59, 60, 74]. These affective vulnerabilities are well-established risk factors for binge-type eating behaviours, including emotional eating and loss-of-control eating, and both clinicians and Autistic individuals themselves reported that sometimes binge eating behaviours are used by this population to regulate their mood and as a self-soothing tool [18, 19, 43].

### Sensory Processing Mechanisms

In contrast, the association between Autism and AN appears to persist even when affective disorders are accounted for [16], suggesting the involvement of additional mechanisms. Qualitative and cross-sectional empirical research has highlighted the potential role of Autism-related traits such as monotropism, exteroceptive sensory sensitivities, and reduced interoceptive awareness in shaping restrictive eating patterns [4, 22, 74]. These processes may contribute to food avoidance, consistent routines around eating, and reduced responsiveness to internal hunger and satiety cues [4, 22].

While sensory sensitivities and interoceptive differences are also reported in Autistic individuals with binge-type EDs, they appear more commonly linked to irregular eating patterns, and less consistently to binge eating [18–20]. Some Autistic and AuDHD adults also describe developing adaptive strategies which may mitigate the impact of these differences on eating behaviour [63, 75].

### Sensory and Reward-Seeking Mechanisms

However, a further pathway that may be particularly relevant to binge-type EDs involves sensory-seeking as well as reward processing, although these constructs are not yet clearly differentiated within the existing literature [18–20, 22, 76]. Qualitative studies have identified sensory-seeking behaviours as a potential driver of bingeing and purging in Autistic individuals, sometimes linked to under-stimulation or attempts to regulate emotional states [18–20, 43]. There are also some reports that these behaviours can become compulsive overtime [18, 43].

This profile may be especially salient in individuals with co-occurring ADHD. Evidence from developmental samples suggests that Autistic-only children tend to present with selective eating (i.e. sensory sensitivities) whereas ADHD-only children present with overeating behaviours. However, AuDHD children present with a combination of selective eating and overeating behaviours [77], with approximately 18.9% of Autistic children displaying this selective overeating profile [78]. However, it is worth noting that not all sensory- or reward-driven eating in Autistic or AuDHD populations are associated with maladaptive eating [63].

Together, these findings suggest that while sensory and interoceptive differences are transdiagnostic, these may play a comparatively smaller role in binge-type EDs than reward-related and affective processes, particularly in the context of co-occurring ADHD.

## Treatment Considerations

### Clinical Presentation

Autism is associated with increased clinical complexity within ED services. For example, in patients with BN, autistic traits are associated with increased ED psychopathology, psychological distress, and functional difficulties [17], as has similarly been found within AN and mixed-diagnosis samples [2, 42, 72, 73, 79, 80].

Autistic individuals with EDs also commonly report difficulties with alexithymia and emotional overwhelm [4, 22]. However, the expression of these difficulties may differ across ED subtypes. AN presentations have been described as often involving more muted or internalised emotional expression, whereas binge-type presentations may involve more overt emotional distress, including heightened impulsivity, self-harm, and suicidality [20]. However, due to the alexithymic nature of this distress, it may present atypically, which can lead to misunderstandings. Autistic individuals with binge-type EDs may be misinterpreted as non-compliant or as presenting with personality-related difficulties, rather than as experiencing distress [20]. Clinicians have also reported uncertainty around how BED presents in Autistic individuals, particularly when conventional diagnostic features such as shame, guilt, or loss of control are described differently or are less clearly articulated [43].

Co-occurring ADHD may further shape clinical presentation in binge-type EDs, contributing to impulsivity, emotional volatility, and executive functioning differences, as well as difficulties maintaining structure and routine, which may also exacerbate binge-purge cycles [20, 37].

### Recognition and Screening within ED Services

Many Autistic individuals present to ED services without Autism having been recognised previously, particularly women, who may experience delayed diagnosis due to masking behaviours and historical biases in diagnostic criteria [79, 81–87]. On average, Autistic individuals receive an Autism diagnosis 6.1 years after their first ED diagnosis [79]. Recognition within ED services is clinically important because Autism is associated with poorer treatment experiences and outcomes, and identifying Autism can help individuals better understand their difficulties and access more appropriate support [5, 34, 81, 88, 89].

Recognition of Autism in restrictive EDs has improved considerably within in recent years. Growing awareness of high co-occurrence rates has led some specialist ED services to introduce routine Autism screening, often the AQ-10, and dedicated care pathways such as PEACE [6, 7, 10, 46]. These developments have helped normalise consideration of Autism within restrictive ED settings, although implementation remains inconsistent across services.

However, Autism remains under-recognised within binge-type ED populations. Clinicians have reported limited perceived relevance of Autism in BED treatment, reflecting broader assumptions that Autism is primarily associated with restrictive EDs, alongside gaps in training and resources [7, 20, 36, 90]. Delayed or missed recognition may contribute to more entrenched ED presentations and limit the effectiveness of standard interventions in this population [20]. Consistent with this, Autistic individuals with BN and BED report that recognising and addressing their Autism within treatment would have improved their understanding of their difficulties and engagement with care [18, 19].

### Treatment Adaptions

There is increasing consensus that ED treatment should be adapted to better meet the needs of Autistic individuals. However, so far there is limited knowledge of treatment options for Autistic individuals with EDs other than AN [11, 36]. Many adaptations developed within AN contexts, such as clear communication, predictable structure, and attention to sensory needs, are also relevant to binge-type EDs [18–20]. However, these alone are unlikely to be sufficient. Greater consideration is needed of factors more salient in binge-type presentations, including sensory-seeking, heightened emotional distress, and reward-driven eating behaviours [7, 20].

Challenges identified regarding Autism and AN, including variability in clinician knowledge, inconsistent implementation of adaptations, and tensions between flexibility and fidelity within manualised treatments, also appear to extend to binge-type ED contexts [7, 20, 30, 35, 91]. As such, calls for clearer clinical guidance, structured care pathways, and more consistent Autism-informed practice appear to be transdiagnostic [7, 20].

## Carer Perspectives

Carer perspectives represent an important but currently under-developed area of Autism-ED research. Carers often play a central role in recognising early signs of eating difficulties, supporting treatment engagement, and navigating complex patterns of relapse and recovery. Within the AN literature, several qualitative studies have explored carer experiences [4, 30–33, 92]. Carers were able to provide a broader, longitudinal perspective on Autistic individuals with AN, highlighting developmental changes such as increasing social difficulties during adolescence, common patterns in the order of Autism and ED diagnoses, and external perspectives on treatment processes, including the importance of clinician-patient alliance and other factors influencing care [4, 30–33, 92].

Carers have also highlighted their own unmet support needs, including a lack of Autism-specific provision within ED services, the need to create independent peer support networks, and the substantial advocacy often required to secure appropriate care. These demands can increase caregiver burden and contribute to major personal, occupational, and financial impacts [4, 30–33, 92].

However, despite this emerging literature in AN and one study including parents of Autistic individuals with EDs more broadly [36], research examining carer perspectives in Autism and binge-type EDs remains extremely limited. This limits understanding of how Autism-related traits and binge-type eating behaviours are experienced within family systems, including how difficulties in emotional regulation, executive functioning, and sensory processing are interpreted and responded to by others. Without these perspectives, existing models risk over-emphasising individual-level mechanisms and under-representing relational and environmental influences.

Clinicians have also noted that parents of Autistic children with binge eating behaviours may adopt highly restrictive monitoring strategies, such as limiting access to certain foods, which can create ongoing family stress and require constant vigilance [43]. These dynamics remain largely unexplored within the current literature.

## Future Research

The current literature on Autism in binge-type EDs remains limited, methodologically heterogeneous, and heavily reliant on trait-based measures. Future research should prioritise the use of gold-standard diagnostic assessments, alongside longitudinal and experimental designs capable of testing proposed affective, sensory, and reward-based pathways. The role of co-occurring ADHD also represents an important avenue for further investigation, particularly in relation to reward sensitivity, impulsivity, and emotional regulation. In addition, there is a need for research translating these mechanisms into clinically actionable treatment adaptations, as existing approaches remain largely derived from AN models. Future research should also prioritise the inclusion of carers alongside Autistic individuals and clinicians in co-produced and qualitative designs, in order to develop a more comprehensive, multi-perspective understanding of binge-type EDs in Autism.

## Conclusions

Overall, current evidence indicates that Autism is over-represented across both restrictive and binge-type EDs, although prevalence appears lower in BN and BED than in AN. Emerging findings suggest both shared and distinct mechanisms underpinning these associations. While sensory and interoceptive differences may operate across ED diagnoses, affective and reward-related processes, particularly in the context of co-occurring ADHD, may play a more prominent role in binge-type presentations. These differences are reflected in clinical presentation, recognition within services, and treatment needs, highlighting the importance of moving beyond an AN-centric model towards a more nuanced, transdiagnostic, and mechanism-informed approach to Autism in EDs. Without such developments, there is a risk that Autism will remain under-recognised and insufficiently addressed within binge-type ED services, limiting both engagement and treatment effectiveness.

## Key References

- Christiansen GB, Petersen LV, Chatwin H, Yilmaz Z, Schendel D, Bulik CM, et al. The role of co-occurring conditions and genetics in the associations of eating disorders with attention-deficit/hyperactivity disorder and autism spectrum disorder. Molecular psychiatry. 2025;30(5):2127–36. https://doi.org/10.1038/s41380-024-02825-w.
  - ○ Large-scale epidemiological and genetic study demonstrating elevated Autism rates in BN while examining the contribution of co-occurring affective disorders.
- Cobbaert L, Millichamp AR, Elwyn R, Silverstein S, Schweizer K, Thomas E, et al. Neurodivergence, intersectionality, and eating disorders: a lived experience-led narrative review. Journal of Eating Disorders. 2024;12(1):187. https://doi.org/10.1186/s40337-024-01126-5.
  - ○ Lived experience-led narrative review providing an important neurodiversity-affirming conceptual framework for understanding Autism, ADHD, and eating disorders.
- Loomes R, Chivers K, Georgeaux‐Healy C, Mandy W, Jewell T. Understanding the Autistic Experience of Restrictive Eating Disorders—A Systematic Review and Qualitative‐Synthesis. European Eating Disorders Review. 2025;33(4):800–14. https://doi.org/10.1002/erv.3181.
  - ○ Comprehensive qualitative synthesis of lived experiences of Autism in restrictive EDs, providing an important conceptual foundation for understanding emerging work in binge-type EDs.
- Makin L, Meyer A, Chubinidze D, Mondelli V, Tchanturia K. Bingeing as an ADHD-Related Strategy: A Qualitative Study of Experiences of Neurodivergent and Potentially Neurodivergent Adults with Bulimic-Spectrum Eating Disorders. Eating and Weight Disorders - Studies on Anorexia, Bulimia and Obesity. 2026;31. https://doi.org/10.1007/s40519-025-01804-6.
  - ○ Qualitative study exploring Autism and ADHD-related experiences in adults with bulimic-spectrum EDs, identifying pathways to binge eating and implications for treatment adaptation.
- Makin L, Mondelli V, Tchanturia K. Escalating Distress and Sensory Seeking: Autism in Binge-Type Eating Disorders from a Clinical Perspective. Autism. 2026.
  - ○ Qualitative study of clinicians' perspectives on recognising and treating Autism in binge-type EDs, highlighting presentation, treatment needs, and the relevance of co-occurring ADHD.
- Makin L, Zesch E, Meyer A, Mondelli V, Tchanturia K. Autism, ADHD, and Their Traits in Adults With Bulimia Nervosa and Binge Eating Disorder: A Scoping Review. European Eating Disorders Review. 2025;33(4):647–65. https://doi.org/10.1002/erv.3177.
  - ○ First scoping review synthesising evidence on Autism and ADHD in adults with BN and BED, highlighting that Autism is over-represented but substantially under-researched in binge-type EDs.
- Meyer A, Makin L, Allen K, Tchanturia K. Prevalence and clinical correlates of autistic traits across eating disorder diagnoses: a retrospective analysis of clinical service data. Nutrients. 2026. https://doi.org/10.3390/nu18172906.
  - ○ Clinical service study demonstrating elevated autistic traits across BN, BED, and AN, and associations with greater ED psychopathology, psychological distress, and functional impairment.
- Nimbley E, Maloney E, Sharpe H, Gillespie-Smith K, Tchanturia K, Duffy F. A mixed method systematic review into the impact of ED treatment in Autistic people and those with high Autistic traits. International Journal of Eating Disorders. 2024. https://doi.org/10.1002/eat.24311.
  - ○ Systematic review demonstrating that Autistic people generally report poorer treatment experiences and outcomes, supporting the need for Autism-informed ED care.
- Saure E, Keski-Rahkonen A. Neurodiversity-affirming eating disorder care: insights into addressing co-occurring autism and eating disorders. Current Opinion in Psychiatry. 2025:10.1097. https://doi.org/10.1097/yco.0000000000001042 .
  - ○ Provides an up-to-date framework for neurodiversity-affirming ED care.
- Tchanturia K, Chubinidze D, Duffy F, Nimbley E, Li Z, Holliday J. Implementation Insights from the PEACE Pathway Across UK Eating Disorder Services. Nutrients. 2025;17(9):1532. https://doi.org/10.3390/nu17091532.
  - ○ Provides practical implementation guidance from the PEACE pathway, demonstrating how Autism-informed adaptations can be embedded within routine ED services.

## Data Availability

No datasets were generated or analysed during the current study.
